# Impact of Storage Duration on the Structural and Functional Properties of Starch in Spicy Strips

**DOI:** 10.3390/foods15050826

**Published:** 2026-03-02

**Authors:** Yujing Ding, Hongling Chao, Xiutian Li, Yang Li, Mingfei Li, Xiaowei Zhang, Shiyuan Miao, Yujie Lu, Dube Nhlanhla Mtelisi

**Affiliations:** 1 School of Grain Science and Technology, Jiangsu University of Science and Technology, Zhenjiang 212000, China; dingyujing321@163.com (Y.D.);; 2 College of Food Science and Engineering, Henan Univeristy of Technology, Zhengzhou 450001, China

**Keywords:** spicy strips, storage duration, starch characteristic and properties

## Abstract

The effects of storage time on the characteristics of starch in spicy strips were investigated. Techniques including differential scanning calorimetry (DSC), thermogravimetric analysis (TGA), and X-ray diffraction (XRD) were employed to analyze the gelatinization properties, thermal characteristics, crystal structure, moisture distribution, and quality changes of spicy strips under different storage periods (0, 60, 120, and 180 days). The results demonstrated that prolonged storage led to a significant decrease in peak viscosity and an increase in setback value, indicating enhanced starch retrogradation. DSC analysis revealed a continuous increase in enthalpy change (ΔH), confirming the formation of more ordered double-helix structures over time. TGA revealed a shift in thermal degradation profiles, indicating changes in component interactions and moisture-binding capacity over storage. XRD patterns showed a clear transition from A-type to V-type crystals and finally to an amorphous state after 180 days. Consequently, solubility, swelling power, and amylose leaching were markedly inhibited, while the retrogradation rate of amylopectin became dominant during long-term storage. These findings provide insights into starch retrogradation mechanisms in complex snack matrices and offer guidance on mitigating quality deterioration during the shelf life of spicy strips.

## 1. Introduction

Spicy strips, a popular snack made primarily from wheat flour or starch through extrusion puffing and seasoning [1], have gained worldwide popularity due to their unique texture, flavor, and convenience, and are widely consumed in Asia and increasingly in Western markets [1]. However, hardening, texture deterioration, and flavor loss during storage significantly limit their shelf life and consumer acceptance [2]. During extrusion processing, raw food materials undergo significant changes in their components, including starch, protein, dietary fiber, lipids, and micronutrients such as vitamins and minerals. The high-temperature, high-pressure, and high-shear environment of the extruder imparts intense thermomechanical energy to the system, leading to the disruption of the semi-crystalline structure of starch and the denaturation of proteins. This process induces starch gelatinization, during which granules dissolve and expand, and new crystal formations may occur [3,4]. At the same time, molecular degradation reactions often occur, generating short-chain dextrins, soluble reducing sugars, and small-molecule wheat substances such as bud sugar [3,4] (a term referring to reducing sugars generated from starch degradation). Specifically, the mechanical shearing force causes the scission of α-1,4 and α-1,6 glycosidic bonds, significantly increasing the proportion of short-chain molecules, which alters the hygroscopicity and subsequent recrystallization kinetics of the matrix. These quality changes are closely related to the physicochemical evolution of starch and proteins during storage [2], yet the interaction mechanisms between these components and their impact on multi-scale structures remain insufficiently understood. In recent years, with the deepening of research on the structure–function relationship of food components during storage, the gelatinization–retrogradation behavior of starch and its synergistic effects have gradually become focal points in food science. In the complex system of spicy strips, the interaction is not merely a physical blend but a molecular reorganization. However, systematic research on the synergistic deterioration mechanisms of starch in the complex processing system of spicy strips remains notably lacking, particularly in areas such as dynamic moisture distribution, molecular force balance, and multi-component interaction networks.

As the main component, starch dictates the texture of spicy strips through its gelatinization behavior, amylose leaching, and retrogradation kinetics. Texture hardening is fundamentally driven by the transition of the starch-water system from a metastable state to a lower-energy crystalline state. Studies have shown that the crystalline structure of starch (such as the transition from A-type to B-type or V-type) undergoes reorganization during storage due to moisture migration and temperature fluctuations, thereby affecting its thermal stability (characterized by thermogravimetric analysis and differential scanning calorimetry) and gelatinization properties (measured using a rapid visco analyzer) [4,5].

Simultaneously, starch’s short-term retrogradation and long-term aging behaviors are controlled by the leaching kinetics of amylose and the rearrangement of amylopectin, processes closely related to moisture distribution (characterized by low-field nuclear magnetic resonance) [6,7]. Within this timeframe, amylose facilitates rapid network formation (short-term), while amylopectin recrystallization dictates the progressive increase in hardness (long-term). Protein molecules, dispersed within the starch matrix, may act as physical barriers or interactive anchors; their competition for water molecules through hydration and the formation of protein–starch complexes via hydrogen bonding significantly modulate the retrogradation rate. Additionally, the reordering of starch molecular chains may compete with proteins for water molecules through hydrogen bonds and hydrophobic interactions, altering the system’s Zeta potential and rheological properties, and further exacerbating quality deterioration. However, the existing research predominantly focuses on the structural evolution of single components [7,8], lacking a systematic analysis of the synergistic mechanisms in the starch–protein–water ternary system [9,10]. The absence of a clear understanding of how the protein–lipid–starch network evolves over a multi-month storage period creates a significant gap in predicting the shelf-life stability of extruded snacks.

Therefore, this study aims to systematically investigate the effects of storage duration on the multi-scale structural and thermal properties of starch in spicy strips. We hypothesize that prolonged storage promotes starch retrogradation, alters crystalline structure from A-type to V-type and eventually to amorphous states, and reduces starch solubility and swelling power due to enhanced molecular reorganization and moisture redistribution.

## 2. Materials and Methods

### 2.1. Materials

The ingredients of the spicy strip blanks provided by the collaborating company included starch, protein, fat, water, and minor components, with contents of 70.12%, 12.68%, 8.62%, and 9.96%, respectively. Upon receipt, they were immediately separated into batches and stored in their original commercial packaging (composite plastic film) under controlled conditions of 25 °C and 60% relative humidity for designated periods (0 days, 60 days, 120 days, and 180 days). The samples stored under commercial conditions were labeled as M0, M2, M4, and M6. Additionally, a fresh spicy strip blank sample (without added oil) was freeze-dried immediately upon receipt and labeled as N0, serving as a control to assess the impact of oil addition and storage on starch properties. Prior to analysis, all samples were freeze-dried by a freeze-dryer (Christ Alpha 1–4 LD plus, Osterode am Harz, Germany, −50 °C, 0.1 mbar), ground using a laboratory mill, and passed through a 60-mesh sieve. All data reported herein are calculated on a dry weight basis unless otherwise stated.

### 2.2. Characterization of Starch Properties

#### 2.2.1. Differential Scanning Calorimetry (DSC)

The thermal properties of starch in spicy strips samples were determined using a DSC-7 analyzer (Perkin Elmer, Norwalk, CT, USA) following the method described by Xie et al. [11]. Approximately 2 mg of sample (dry basis) was weighed in an aluminum pan, and distilled water was added to achieve a starch-to-water ratio of 1:4 (*w*/*w*). After sealing, the sample was equilibrated at 25 °C for 12 h. It was then heated from 20 °C to 120 °C at a rate of 10 °C/min under a nitrogen atmosphere (flow rate: 20 mL/min). The onset (T_0_), peak (T_P_), conclusion (T_C_) temperatures, and enthalpy change (ΔH) were calculated by integrating the endothermic peak area using the instrument’s Pyris Software (PerkinElmer 10.1.0.412). All measurements were performed in triplicate. The onset (T_0_), peak (T_P_), conclusion (T_C_) temperatures, and enthalpy change (ΔH) were calculated using Pyris Software (PerkinElmer) by integrating the endothermic peak area. All measurements were performed in triplicate.

#### 2.2.2. Thermogravimetric Analysis (TGA)

To evaluate thermal stability, TGA was performed. All measurements were performed in triplicate. Thermogravimetric analysis was performed using a PerkinElmer Pyris 1 system. Samples (9–11 mg) were heated from 30 °C to 600 °C at 10 °C/min under nitrogen flow (20 mL/min). The mass loss (%) and derivative thermogravimetric (DTG) peaks were analyzed using Pyris Thermal Analysis software (10.1.0.412). The degradation temperatures and mass loss rates for each stage were recorded.

#### 2.2.3. Pasting Properties

The freeze-dried spicy strip powders (3.0 g, 14 g/100 g moisture basis) were directly weighed in the canister of an RVA (RVA-3; Recorded in centipoise (cP) using Software Thermocline for Windows (11.2). Newport Scientific, Narrabeen, Australia), and distilled water was added to obtain a sample weight of 28.0 g. The slurry was then manually homogenized using a plastic paddle to avoid lump formation before the RVA analysis. The slurry was heated from 50 °C to 95 °C at a rate of 12 °C/min and maintained at 95 °C for 2.5 min. It was then cooled to 50 °C at the same rate and held at 50 °C for 2 min. Recorded in centipoise (cP) using Software Thermocline for Windows. The pasting temperature and peak, breakdown, and setback viscosities were recorded [6].

#### 2.2.4. Solubility and Swelling Properties

Weigh 0.2000 g of the sample (dry basis) with a precision of 0.0001 g and prepare a 10 mL starch milk solution with a mass fraction of 2%. Stir the solution in a water bath at 80 °C and 90 °C for 30 min, respectively. After cooling to room temperature, centrifuge the solution at 3000 rpm (approximately × *g*) for 15 min. Pour the supernatant into an aluminum box and dry it in an oven at 105 °C for 4 h, then weigh to obtain the mass of water-soluble starch. The precipitate is the swollen starch. All measurements were performed in triplicate (*n* = 3).

The supernatant was decanted into a pre-weighed aluminum dish and dried at 105 °C to constant weight (W1) to determine soluble solids. The wet precipitate (Wp) was weighed to determine swelling power. Solubility (S) and swelling power (SP) were calculated using the following equations:

S(%) = W1/Ws × 100(1)

SP(g/g) = Wp/[Ws × (1 − S/100)](2)

#### 2.2.5. Degree of Gelatinization

The degree of gelatinization (DG) was determined based on the differential binding capacity of iodine to gelatinized versus native (non-gelatinized) starch [6]. Centrifuge the prepared starch paste at 15,000× *g* for 20 min to collect the supernatant. Mix 1 mL of the supernatant with 6 mL of 0.33 M NaOH solution and heat at 95 °C for 30 min. After cooling to room temperature, adjust the pH of the mixed solution to 5.5 using a 0.5% trichloroacetic acid solution. Then, add 1.0 mL of 0.2% (*w*/*v*) I_2_-KI solution to initiate the reaction. Allow the reaction to proceed in the dark for 20 min and measure the absorbance of the sample at 620 nm.

The gelatinization degree was determined using the glucose amylase method [6]. Precisely weigh 1.00 g (dry basis) of the sample, which has been sieved through a 60-mesh sieve (to remove clumped flour), into flasks A1 and A2, and prepare flask B as a blank control. Add 50 mL of distilled water to each of the aforementioned flasks and shake well to mix. Subject flask A1 to a boiling water bath for gelatinization for 20 min, then rapidly cool it to room temperature using cold water. Subsequently, add 5 mL of a 2% (m/V) glucose amylase solution (freshly prepared) to flasks A1, A2, and B, and incubate in a constant temperature water bath at 50 °C with shaking for 1 h. Immediately after, add 2 mL of 1 mol/L hydrochloric acid to halt the saccharification process, cool to room temperature, dilute to a final volume of 100 mL, and filter to obtain the test solution.

Measure 10 mL of each test solution into three separate 100 mL iodine flasks. Sequentially add 10 mL of 0.1 mol/L iodine solution and 18 mL of 0.1 mol/L NaOH solution to each flask. Seal the flasks and place them in a dark location to stand for 15 min. Afterward, quickly add 2 mL of 10% (m/m) H_2_SO_4_ to each flask. Titrate the mixture with 0.1 mol/L Na_2_S_2_O_3_ solution until the solution becomes colorless and record the volume of Na_2_S_2_O_3_ solution consumed.

DG(%) = (V0 − V2)/(V0 − V1) × 100(3)

In the formula: V0,V1 and V2 represent the volumes (in milliliters) of the Na_2_S_2_O_3_ solution consumed by the blank, gelatinized, and non-gelatinized samples, respectively.

#### 2.2.6. Leaching of Amylose and Retrogradation Rate of Starch

Take 1 g of the sample and add an appropriate amount of distilled water to prepare a starch slurry with a mass fraction of 20%. After thoroughly mixing, gelatinize the slurry by stirring in a constant temperature water bath at 90 °C for 10 min, followed by high-pressure treatment in an autoclave at 0.1 MPa for 20 min. Allow the sample to cool slowly to room temperature, then place it at 4 °C for retrogradation. Retrogradation times for the mixture are 0 h, 2 h, 3 h, 4 h, 5 h, 8 h, 12 h, 14 h, and 16 h.

For the retrograded samples of the amylose–protein mixture at different time intervals, perform enzymatic hydrolysis by adding 100 U/g of neutral protease and incubating in a constant temperature water bath at 50 °C for 1 h. Then, add an appropriate amount of α-thermostable amylase and incubate in a constant temperature water bath at 90 °C for 1 h.

For the retrograded samples of the amylopectin–protein mixture at different time intervals, perform enzymatic hydrolysis by adding 100 U/g of neutral protease and incubating in a constant temperature water bath at 50 °C for 1 h. Then, add an appropriate amount of medium-temperature amylase and incubate in a constant temperature water bath at 70 °C for 1 h.

After enzymatic hydrolysis, wash the mixed samples several times with an appropriate amount of distilled water by centrifugation until the solution becomes colorless. Transfer the samples to clean Petri dishes and dry them in a blast drying oven. Weigh the Petri dishes and calculate the retrogradation rate using the following formula:

Retrogradation Rate (%) = (m/m0) × 100%(4)

In the formula: m represents the weight of the purified retrograded sample, and m0 represents the weight of the sample before retrogradation.

#### 2.2.7. XRD-Ray

The crystal types and intensities of the sample powders prepared after different storage periods were determined using an X-ray diffractometer (D8 Advance A25, German Bruker Company, Karlsruhe, Germany). A 0.5 g sample powder was evenly spread on a glass plate, flattened using a slip, and then placed into the X-ray diffractometer for testing at room temperature. The testing conditions were as follows: tube voltage of 45 kV, tube current of 40 mA, scanning range of 5~60°, and scanning speed of 2°/min [12].

### 2.3. Statistical Analysis

All experiments were performed in triplicate. Results are expressed as the mean ± standard deviation of triplicate experiments. Data were analyzed by one-way analysis of variance (ANOVA); statistical significance was defined at *p* < 0.05, followed by Duncan’s multiple range test using SPSS 16.0 Statistical Software Program (SPSS Incorporated, Chicago, IL, USA).

## 3. Results and Discussion

All analyses were performed on freeze-dried powders to standardize moisture content and focus on structural changes. It should be noted that the samples were freeze-dried prior to analysis, not before storage. While this approach provides precise mechanistic insights, it should be considered that the absence of plasticizing water during analysis may accentuate certain thermal transitions compared to the native hydrated state during storage.

### 3.1. Pasting Properties

The gelatinization of wheat flour is primarily attributed to the gelatinization process of wheat starch. The process of starch gelatinization and retrogradation can be described as follows: when starch is mixed with water and the sample’s temperature increases, the native starch granules undergo disruption and disintegration [13]. Table 1 presents the starch gelatinization characteristics of spicy strips during storage. As shown in the table, the peak viscosity, through viscosity, breakdown value, and setback value of the freeze-dried spicy strip base (N0) are higher than those of the freeze-dried spicy strip bases (M0, M2, M4, M6). This is primarily attributed to the addition of oil to the spicy strips, which delays starch retrogradation [14]. Additionally, the formation of lipid–starch complexes hinders the entry of water molecules into the starch granules, slowing down the leaching of amylose and thereby reducing gelatinization viscosity. As storage time increases, the peak viscosity of the freeze-dried spicy strip powder gradually decreases. This phenomenon can be explained from two perspectives: (1) During gelatinization, gluten proteins with fibrous and spherical structures adhere to the surface of starch granules. Simultaneously, lipid–starch–gluten complexes are formed through covalent or non-covalent bonds in the spicy strips. (2). It is hypothesized that lipid–starch–gluten complexes might be formed via non-covalent interactions (e.g., hydrogen bonding and hydrophobic interactions), as suggested by previous studies on extruded flour systems. Moisture loss during storage affects the transport of adequate water and the concentration of starch in the paste system [9]. Combined with the results of moisture distribution, the bound water and free water in M6 decrease, leading to a lower relative concentration of starch during gelatinization and lower peak and final viscosities. Regarding Peak Viscosity: The peak viscosity of the spicy strip powder decreased significantly with increased storage time, dropping from 108 cP at M0 to 85 cP at M6 (*p* < 0.05). Regarding Setback Value: The setback value increased gradually from 22.50 cP at M0 to 35.50 cP at M6, indicating an enhanced retrogradation trend. Regarding Enthalpy Change: DSC analysis revealed that the enthalpy change (ΔH) increased with prolonged storage time (from 9.18 J/g at M0 to 13.24 J/g at M6), confirming the formation of ordered structures.

Furthermore, the thermal properties of lipids and gluten influence energy transfer during starch gelatinization, thereby affecting the swelling of starch granules. The setback value reflects starch’s retrogradation characteristics, indicating the amorphous region’s recrystallization and the reassociation of amylose–amylose molecules. A higher setback value suggests a greater tendency for retrogradation. The table shows that the setback value of the spicy strip base (N0) is significantly higher than that of the freeze-dried spicy strip powder. Moreover, the setback value gradually increases with prolonged storage time. The fresh freeze-dried spicy strip powder (M0) exhibits the lowest setback value (22.50 cP), indicating that fresh spicy strip samples are the least prone to retrogradation. This may be due to the sufficient contact between oil molecules and amylose molecules, reducing the opportunity for starch molecules to interact and thereby minimizing molecular rearrangement and aggregation. Adding fatty acids increases the distance between starch molecular chains, reduces the hydrogen bond content between starch molecules, and inhibits the formation of double-helix structures and the recrystallization of amylose. Additionally, fatty acids contribute to a denser and more stable structure in the starch–gluten protein system, enhancing water retention and slowing down the retrogradation rate [15].

### 3.2. Solubility and Swelling Properties

**Figure 1:**
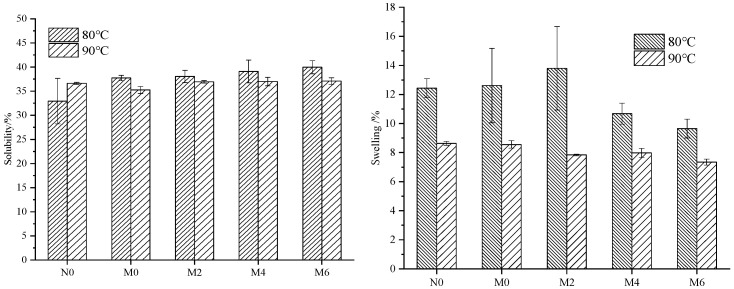
Changes in solubility and swelling power during storage of spicy strips.

Starch’s solubility and swelling power in wheat flour reflect the interactions between starch granules and molecules [10]. Solubility primarily depends on the binding capacity between soluble starch and water molecules. Soluble starch (amylose) contains hydrophilic groups that readily bind with water molecules, while insoluble starch molecules (amylopectin) are stabilized by extensive hydrogen bonds, making their structure more resistant to dissolution. When these insoluble starch molecules interact with water, they exhibit swelling behavior, which is swelling power. The results of solubility and swelling power during the storage of spicy strips are illustrated in Figure 1.

In Figure 1, it can be observed that the solubility of freeze-dried spicy strip powder at 80 °C and 90 °C shows no significant trend with prolonged storage time. However, the swelling power of samples M0 and M2 at 80 °C is significantly higher than that of other samples. Additionally, the swelling power of freeze-dried spicy strip flour decreases considerably after 4 months of storage. This phenomenon may be attributed to the fact that, after 4 months, more amylopectin molecules undergo rearrangement and aggregation through hydrogen bonding, leading to the reformation of double-helix structures and the retrogradation and aging of amylopectin [16]. As a result, water molecules find it more challenging to penetrate the starch molecules and bind with amylopectin, causing a significant reduction in swelling power.

### 3.3. Thermal Properties (DSC)

Differential scanning calorimetry (DSC) is a thermal analysis technique that plots temperature on the x-axis and the rate of heat absorption or release by the sample on the y-axis, recording the relationship between the sample, reference material, and temperature [17]. In gelatinized starch systems, amylose rapidly retrogrades as the temperature decreases, forming dense and stable crystals. These crystals typically melt at around 115 °C. In contrast, amylopectin, with its highly branched structure, is less prone to retrogradation than amylose. The melting of crystals requires heat absorption, and under specific temperature conditions, the crystals formed by starch retrogradation melt, resulting in an endothermic peak in the DSC curve. Generally, the greater the degree of starch retrogradation, the larger the peak area and enthalpy value of the endothermic peak in the DSC curve.

Table 2 presents the gelatinization temperatures (T_0_, T_P_, T_C_), the temperature range (T_C_–T_0_), and the enthalpy change (ΔH) of freeze-dried spicy strip flour. The table shows the enthalpy change (ΔH) increases with prolonged storage time [18], suggesting that the degree of starch retrogradation in spicy strips becomes more pronounced over time. This phenomenon may be attributed to amylopectin being associated with long-term retrogradation, while amylose is linked to short-term retrogradation. Additionally, the enthalpy change (ΔH) reflects the formation of double-helix structures. An increase in ΔH indicates more double-helix structures, implying enhanced thermal stability [19].

The continuous increase in ΔH (Table 2), indicative of more ordered double-helix formation, aligns with the observed reduction in peak viscosity (Table 1) and supports the progression of starch retrogradation during storage.

### 3.4. Thermogravimetric Analysis (TGA/DTG)

**Figure 2:**
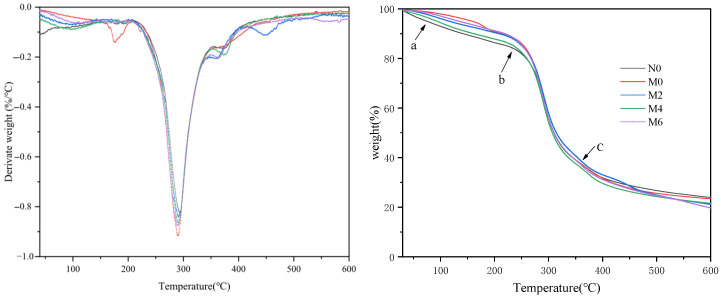
Thermal degradation profiles (TGA/DTG) of spicy strips during storage. a, the turning point of mass loss in the stage 1 of the sample; b, the turning point of rapid mass loss in the stage 2 of the sample; c, the turning point of minor mass loss in the stage 3 of the sample.

To explore the thermal decomposition process of starch in the presence of gluten, thermogravimetric analysis (TGA and DTG) was conducted on mixed powders of starch and wheat gluten with varying gluten strengths [20]. The results are shown in Figure 2.

From the TGA curves, it can be observed that all samples exhibit three stages of mass decomposition: Stage 1 (60–150 °C): The initial mass loss in samples (Figure 2) at 60–150 °C was due to the free and bonded water loss with the increasing temperature. Stage 2 (250–350 °C): A rapid mass loss at higher temperatures was primarily due to the wheat flour’s degradation of lipids, proteins, and starch molecules. This stage is characterized by the breakdown of hydroxyl groups on the glucose rings of starch, leading to the formation of water molecules and the cleavage of bonds such as C-C-H, C-O, and C-C within starch molecules. Simultaneously, covalent peptide bonds in protein amino acid residues and bonds such as S-S, O-N, and O-O within protein molecules are broken [21,22]. Stage 3 (390–600 °C): A minor mass loss at even higher temperatures resulted from the carbonization of decomposition products. The corresponding DTG (first derivative of mass concerning temperature) curves also show three mass loss peaks. However, the peak for the third stage is not particularly prominent due to the relatively small mass loss. The thermal degradation temperatures and mass loss rates for the first and second stages of the mixtures are summarized in Table 3.

As shown in Figure 2 and Table 3, it can be observed that the freeze-dried spicy strip flour (without oil) exhibits a water evaporation temperature of 52.72 °C in the first stage, which is significantly lower than that of the freeze-dried spicy strip powder (M0, M2, M4, M6). This indicates that the spicy strips base without oil has the weakest ability to bind water molecules. Additionally, the mass loss in this stage for M4 and M6 freeze-dried spicy strips powders is 4.32% and 4.72%, respectively, significantly higher than that of M0 and M2 powders (3.20% and 1.85%, respectively). The M0 sample shows the highest peak temperature for water evaporation and the lowest mass loss, suggesting that fresh spicy strips can effectively bind water molecules. Furthermore, the lipid–gluten molecular structure can significantly prevent moisture loss.

In the temperature range of 200–600 °C, the DTG curves exhibit significantly different trends, indicating varying degrees of thermal decomposition of the materials. The DTG curve of the M0 sample shows the highest peak, suggesting that proteins, lipids, and starch undergo the fastest mass loss within this temperature range. The peak decomposition temperature for the M0 sample occurs at 291.19 °C, with a mass loss of 36.67% during this stage. This stage of decomposition indicates that starch molecules are gradually losing their ordered crystalline regions, which are being disrupted. Long-chain starch molecules break down into shorter chains, significantly reducing the degree of polymerization. This also suggests that the starch has relatively poor thermal stability, consistent with the enthalpy change (ΔH) results obtained from DSC analysis.

### 3.5. Gelatinization Degree

**Figure 3:**
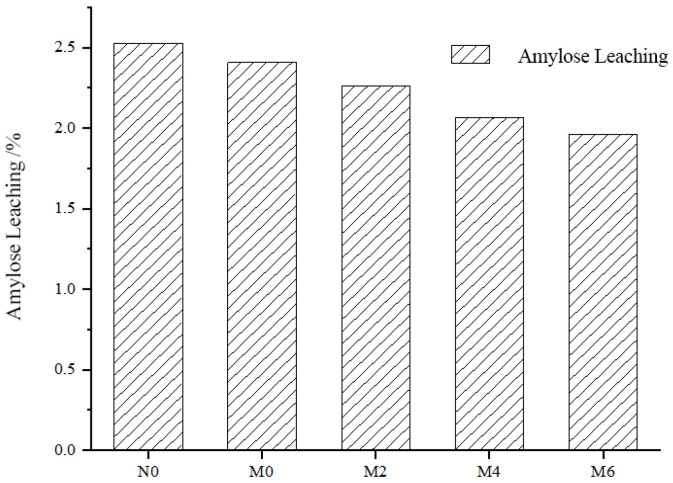
Amylose leaching during storage of spicy strips.

The gelatinization degree quantitatively represents the extent to which starch granules have undergone gelatinization. The gelatinization degrees of freeze-dried powders during the storage of spicy strips are presented in Table 3. As shown in the table, the freeze-dried spicy strip base powder (without oil) exhibits the highest degree of gelatinization (81.10%) compared to the freeze-dried spicy strip powder (with oil). Additionally, the degree of gelatinization significantly decreases with prolonged storage time. This suggests that the addition of oil and the progression of storage affect the gelatinization behavior of starch, likely due to changes in starch structure and interactions with other components such as lipids and proteins. The oil forms a physical barrier on the surface of starch granules, limiting water penetration into the granules, thereby inhibiting swelling and gelatinization. This is consistent with the observation that the oil-free base (N0) exhibited the highest gelatinization degree (81.10%). Association with Amylose Leaching: The formation of lipid–amylose complexes further impedes the leaching of amylose (Figure 3), leading to restricted gelatinization during thermal processing. Storage-Induced Structural Reordering: As storage time increased, the rise in DSC enthalpy (ΔH) from 9.18 to 13.24 J/g reflects the reordering of starch molecular chains and an increase in double-helical structures. This storage-induced molecular ordering enhances the structural stability of the starch, making it more resistant to subsequent gelatinization processes and contributing to a significant decrease in the gelatinization degree.

### 3.6. Analysis of Amylose Leaching and Starch Retrogradation Rate

The amount of amylose leaching reflects the hydration capacity of starch under heating conditions and serves as an essential reference for its processing characteristics [8]. The results of amylose leaching in freeze-dried powders during the storage of spicy strips are shown in Figure 3. As illustrated in the figure, the amount of amylose leaching significantly decreases with prolonged storage time, while the retrogradation value gradually increases. This phenomenon can be attributed to two main reasons: Firstly, formation of lipid–protein–starch complexes: Lipids and proteins form complexes with starch, covering the surface of starch granules and limiting the entry of water molecules into the granules. This hinders the leaching of amylose. Secondly, the role of amylopectin in retrogradation: Starch retrogradation is primarily driven by the reorganization of amylopectin rather than amylose as storage time increases [7].

**Figure 4:**
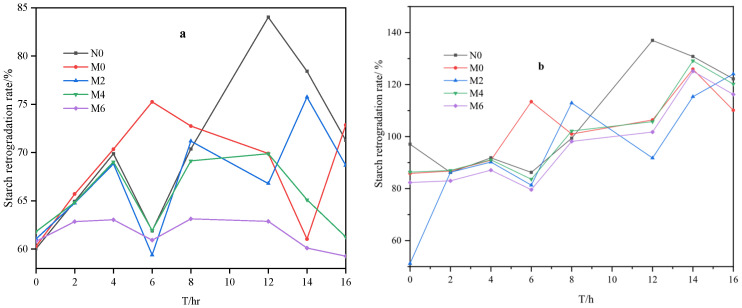
Retrogradation rates of amylose (**a**) and amylopectin (**b**).

As shown in Figure 3 and Figure 4, the retrogradation rate of amylose gradually decreases over time, while the retrogradation rate of amylopectin generally indicates an increasing trend. This further supports the idea that amylopectin plays a dominant role in the retrogradation process during prolonged storage.

### 3.7. XRD

**Figure 5:**
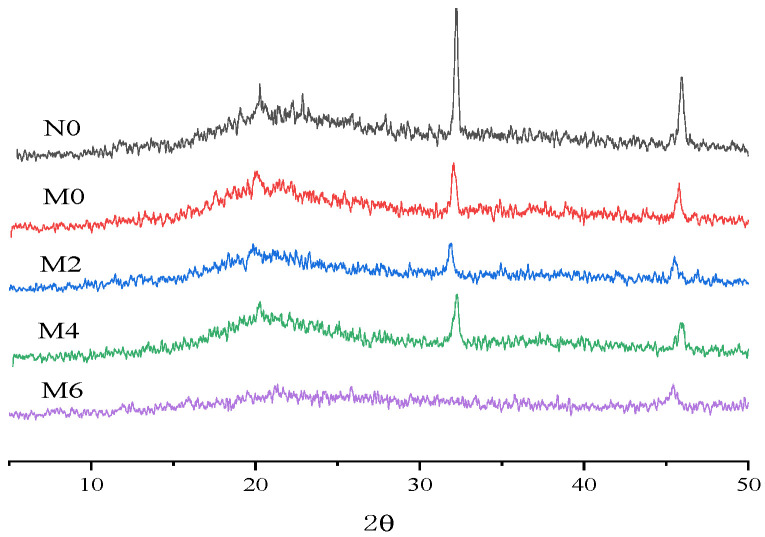
X-ray diffraction (XRD) curves during storage of spicy strips.

Starch retrogradation refers to the process in which gelatinized starch molecules gradually realign in an ordered manner with the participation of water molecules. Hydrogen bonds between adjacent molecules are restored, forming a dense, highly crystalline structure. X-ray diffraction (XRD) is one of the most commonly used methods for determining the crystallinity of starch granules. In the XRD pattern of starch, diffraction peaks correspond to crystalline regions, while diffuse peaks represent amorphous and subcrystalline regions. The intensity of a diffraction peak for a specific phase in a polycrystalline mixture is proportional to the content of that phase in the mixture, allowing us to reflect the crystallinity of starch. Typically, a higher and narrower diffraction peak indicates increased crystalline content and a more substantial degree of retrogradation. The XRD patterns of freeze-dried flour during the storage of spicy strips are shown in Figure 5.

Lipids form stable complexes with amylose molecules, inhibiting the rearrangement of amylose and delaying the characteristic peaks of amylose recrystallization. As shown in Figure 5, the distinctive peaks of the starch–gluten protein system appear near 17° and 20°, indicating that the starch structure is primarily of the A-type. The diffraction peak near 20° is attributed to the formation of V-type complexes between starch and endogenous lipids within the granules [23]. The characteristic diffraction peaks for N0, M0, M2, and M4 samples appear at 2θvalues of 21°, 33°, and 46°, with the crystalline structure of starch composed of both diffraction peaks and diffuse peaks. In contrast, the M6 sample exhibits predominantly diffuse peaks, suggesting a transition from crystalline to amorphous regions in the starch. Additionally, the diffraction peaks at 21° and 33° disappear, indicating that the crystalline structure of the spicy strips changes with prolonged storage time [24].

The transition from A-type to V-type and finally to a more amorphous pattern (Figure 5) correlates with the increased ΔH values from DSC. This suggests that, while long-term storage promotes overall molecular reordering (increased ΔH), the crystalline long-range order may eventually become disrupted, leading to the observed amorphous XRD pattern in the M6 sample.

## 4. Conclusions

This study demonstrates that storage time significantly affects the structural and thermal properties of starch in spicy strips. Storage time acts as the primary driver for the system’s thermodynamic evolution, transitioning from a disordered post-extrusion state to a more ordered but texture-compromised state. Prolonged storage promotes starch retrogradation, as evidenced by increased ΔH, setback viscosity, and amylopectin retrogradation rate. The continuous increase in enthalpy (ΔH) reflects the gradual re-establishment of double helices and the consolidation of the crystalline lattice over time. XRD analysis revealed a gradual transition from A-type to V-type crystals, ultimately leading to an amorphous structure after 180 days. This structural evolution follows a time-dependent kinetic path: the initial 30–90 days are characterized by rapid moisture redistribution and amylose-driven reorganization, followed by a slower, progressive development of V-type complexes (amylose–lipid complexes), and eventually, a potential loss of long-range order as the matrix becomes excessively dehydrated and rigidified into an amorphous state. Oil addition initially inhibits retrogradation but does not prevent long-term structural deterioration. The inhibitory effect of oil is likely due to the formation of a physical film and amylose–lipid complexes in the early stages, yet these barriers are insufficient to arrest the long-term thermodynamic drive of amylopectin recrystallization. These findings provide insights into the mechanisms of texture hardening in spicy strips and suggest that controlling storage conditions and optimizing formulation could extend shelf life. In summary, the quality deterioration of spicy strips is a progressive, non-linear process where the first 120 days represent a critical period of structural transformation, after which the material properties tend to stabilize in a highly retrograded, amorphous state.

## Figures and Tables

**Table 1:** Results of starch gelatinization characteristics (RVA) during storage of spicy strips.

Sample	Peak Viscosity (cP)	Trough Viscosity (cP)	Breakdown (cP)	Final Viscosity (cP)	Setback (cP)	Pasting Time (min)
N0	251.50 ± 9.19 ^a^	212.50 ± 6.36 ^a^	39.00 ± 2.83 ^a^	265.50 ± 12.02 ^a^	53.00 ± 5.66 ^a^	4.70 ± 0.05 ^b^
M0	108.00 ± 2.83 ^b^	104.50 ± 3.54 ^b^	3.50 ± 0.71 ^b^	127.00 ± 5.66 ^b^	22.50 ± 2.12 ^d^	6.87 ± 0.00 ^a^
M2	105.00 ± 2.83 ^b^	100.50 ± 3.54 ^b^	4.50 ± 0.71 ^b^	132.50 ± 4.95 ^b^	32.00 ± 1.41 ^c^	6.93 ± 0.00 ^a^
M4	91.00 ± 1.41 ^c^	88.50 ± 0.71 ^c^	2.50 ± 0.71 ^b^	119.00 ± 4.24 ^b^	30.50 ± 3.54 ^c^	6.70 ± 0.05 ^a^
M6	85.00 ± 1.08 ^c^	86.00 ± 1.15 ^c^	1.50 ± 0.25 ^b^	98.00 ± 3.65 ^c^	35.50 ± 0.15 ^b^	6.80 ± 0.00 ^a^

N0 represents the freeze-dried powder of the spicy strip base; M0, M2, M4, and M6 represent the freeze-dried powders of spicy strips stored for 0, 2, 4, and 6 months, respectively. The results were reported as the mean ± standard deviation. Different lower-case letters in the same column indicate significant differences (*p* < 0.05).

**Table 2:** Thermodynamic parameters during storage of spicy strips.

Sample	T_0_/°C	T_P_/°C	T_C_/°C	ΔH (J/g)	T_C_–T_0_
N0	60.50 ± 0.23 ^c^	89.60 ± 0.13 ^ab^	91.50 ± 0.33 ^b^	6.80 ± 0.41 ^c^	31.00 ± 0.25 ^a^
M0	82.60 ± 0.31 ^a^	97.09 ± 0.22 ^a^	101.90 ± 0.30 ^a^	9.18 ± 0.26 ^b^	19.30 ± 0.21 ^c^
M2	73.50 ± 0.20 ^b^	86.43 ± 0.26 ^b^	101.30 ± 0.31 ^a^	8.96 ± 0.32 ^b^	27.80 ± 0.27 ^b^
M4	84.01 ± 0.13 ^a^	87.12 ± 0.27 ^b^	102.56 ± 0.37 ^a^	10.63 ± 0.28 ^ab^	18.55 ± 0.13 ^c^
M6	86.05 ± 0.16 ^a^	95.25 ± 0.43 ^a^	105.60 ± 0.53 ^a^	13.24 ± 0.61 ^a^	19.55 ± 0.21 ^c^

The results were reported as the mean ± standard deviation. Different lower-case letters in the same column indicate significant differences (*p* < 0.05). T_0_: onset temperature; T_P_: peak temperature; T_C_: conclusion temperature; ΔH: enthalpy change; T_C_–T_0_: gelatinization temperature range.

**Table 3:** Thermal degradation temperatures, mass loss rates and gelatinization degree of freeze-dried powder during storage of spicy strips.

Sample	Degradation Temperature (°C)	Mass Loss (%)	Degradation Temperature (°C)	Mass Loss (%)	Gelatinization Degree (%)
N0	52.72 ± 0.71 ^b^	3.09 ± 0.11 ^a^	293.83 ± 1.37 ^a^	39.12 ± 0.76 ^a^	81.10 ± 2.27 ^a^
M0	95.13 ± 0.56 ^a^	1.85 ± 0.12 ^b^	291.19 ± 1.62 ^a^	36.67 ± 0.54 ^a^	67.05 ± 3.10 ^b^
M2	89.56 ± 0.41 ^a^	3.20 ± 0.20 ^a^	294.55 ± 2.01 ^a^	37.70 ± 0.38 ^a^	62.18 ± 1.65 ^c^
M4	90.56 ± 0.62 ^a^	4.73 ± 0.13 ^a^	291.91 ± 1.24 ^a^	38.23 ± 0.49 ^a^	56.32 ± 2.25 ^d^
M6	90.36 ± 0.52 ^a^	4.32 ± 0.31 ^a^	290.24 ± 1.32 ^a^	36.13 ± 0.46 ^a^	48.68 ± 1.35 ^e^

The results were reported as the mean ± standard deviation. Different lower-case letters in the same column indicate significant differences (*p* < 0.05).

## Data Availability

The original contributions presented in this study are included in the article. Further inquiries can be directed to the corresponding author.

## Abbreviations

The following abbreviations are used in this manuscript:

DSC	Differential Scanning Calorimetry
TGA	Thermogravimetric Analysis
XRD	X-Ray Diffraction
ΔH	Enthalpy change
